# *AIP1*, Encoding the Small Subunit of Acetolactate Synthase, Is Partially Responsible for Resistance to Hypoxic Stress in *Arabidopsis thaliana*

**DOI:** 10.3390/plants10112251

**Published:** 2021-10-22

**Authors:** Geunmuk Im, Dongsu Choi

**Affiliations:** Department of Biology, Kunsan National University, Gunsan-si 54150, Korea; areas12@kunsan.ac.kr

**Keywords:** ALS, BCAA, low oxygen, flooding, *AIP1*

## Abstract

Flooding is a significant stress to land plants, depriving them of essential oxygen. Plants have evolved diverse strategies with variable success to survive flooding. Similar strategies have been described in organisms from other kingdoms. Several fungal species can successfully survive a low-oxygen environment by increasing their branched-chain amino acid (BCAA) contents. BCAAs may act as alternative electron acceptors in the respiratory chain under an oxygen-limited environment. The key and first enzyme for BCAA biosynthesis is acetolactate synthase (ALS). We identified two homologous genes encoding the small subunit of ALS in Arabidopsis (*Arabidopsis thaliana*). We determined that *ALS INTERACTING PROTEIN1* (*AIP1*), which encodes the small subunit of ALS, is strongly expressed in all organs and highly expressed under submergence and low-oxygen stresses. We also showed that the overexpression of *AIP1* confers tolerance to low-oxygen stress. These results indicate that ALS may play an essential role under prolonged flooding or oxygen deficiency in Arabidopsis.

## 1. Introduction

Due to the rapid progression of climate change, crop productivity has recently been declining in harsh environments [1]. As the world population continues to increase, a stable food supply is and will be a critical issue. By 2100, the average global temperature is predicted to rise by 2 °C, which will affect the climate in unprecedented ways [2]. Climate change is also responsible for sea levels rising and the flooding of farmlands, which will adversely influence agriculture and crop yields [3]. Frequent and prolonged flooding significantly reduces production of major staple crops such as rice (*Oryza sativa*), wheat (*Triticum aestivum*), and maize (*Zea mays*), which will threaten food security in the near future [1].

Flooding causes the depletion of oxygen inside plant cells, which blocks oxidative phosphorylation, dramatically reducing energy production and leading to severe hypoxic stress. Some plant species survive flooding by adopting special strategies, such as the formation of specialized tissue called aerenchyma that creates pockets of air [4,5]. Among such species, deepwater rice shows unique behaviors under prolonged flooding since it can escape complete submergence by promoting rapid stem growth to acquire enough oxygen supply from leaves that reach above the water line. By contrast, other flooding-tolerant species employ a breath-holding strategy, greatly reducing their metabolism until floodwaters recede. Plant adaptive strategies for flooding stress are diverse, complex, and remain mostly unexplained [4,6]. In recent years, various studies have focused on oxygen-sensing mechanisms that detect oxygen deficiency caused by flooding [7,8,9] and are the underlying transcriptional regulation. However, there are relatively few studies focusing on the factors that ultimately induce plant responses to hypoxia. Hypoxia and flooding induce changes in various metabolic processes [10,11,12], so certain enzymes and metabolites may play an important role in plant adaptive responses to hypoxia or flooding. Among diverse metabolic adaptations, the accumulation of amino acids can be essential to the survival of plants under the hypoxic conditions caused by floods [13,14,15,16].

In a microarray-based study using deepwater rice [11], we previously determined that the submergence-responsive gene (Os02g39570) encodes the putative regulatory subunit of acetolactate synthase (ALS) responsible for the biosynthesis of a certain group of amino acids. ALS has been the focus of studies pertaining to herbicide resistance. Since several amino acid biosynthetic pathways are targets of some herbicides, the metabolism of certain amino acids has been intensively studied [17,18].

Amino acids are the building blocks of protein biosynthesis, constituting a major organic form of transported nitrogen in plants by lying at the crossroads between carbon and nitrogen metabolism. They also participate in essential physiological processes, as they are the precursors of many plant secondary metabolites such as lignin, phytohormones, and flavonoids [19,20]. Of all essential amino acids, valine (Val), leucine (Leu), and isoleucine (Ile) have side chains and are collectively referred to as branched-chain amino acids (BCAAs) [21]. BCAAs are classified according to the small branching hydrocarbon residues responsible for their aliphatic character [21]. Plant BCAAs are essential compounds: They play an essential role in the biosynthesis of various secondary metabolites, in addition to their function as protein constituents [21,22]. Val and Ile are synthesized via two parallel pathways involving four common enzymes, which catalyze different products in the presence of different substrates [23]. ALS, the first common enzyme in the pathway, uses two pyruvate molecules to form acetolactate, leading to Val and Leu biosynthesis. Alternatively, the enzyme uses one pyruvate and one α-ketobutyrate substrate molecule to generate acetohydroxybutyrate as a precursor for Ile biosynthesis [23]. ALS has two subunits: a catalytic subunit and a regulatory subunit [24,25]. The regulatory subunit greatly enhances the reactivity of the catalytic subunit and is necessary for feedback inhibition by BCAAs [18,26,27,28].

Some fungi adapt to hypoxic environments through biosynthesis of BCAAs [29]. In this case, the fungus produces BCAAs to re-oxidize NAD(P)H and ALS strongly influences BCAA production in the hypoxic state [29]. Thus, as a result of the activation of ALS, the fungus gains an energy source in low-oxygen conditions that contributes to its survival. However, no hypoxic studies have been conducted on the genes encoding ALS regulatory subunits in seed plants.

In this study, we characterized two Arabidopsis (*Arabidopsis thaliana*) genes (*ALS INTERACTING PROTEIN1* [*AIP1*] and *AIP3*) orthologous to the rice ALS small subunit genes [27]. *AIP1* and *AIP3* encode ALS regulatory subunits and have diverse biological functions. However, their roles in hypoxia responses have not been studied in land plants. To understand their possible biological roles during hypoxia, we generated transgenic Arabidopsis plants overexpressing each gene. In this research, we provide the first report on the possible role of ALS in the low-oxygen response of Arabidopsis.

## 2. Results

### 2.1. Organ-Specific Expression Patterns of AIP1 and AIP3 Genes

**Figure 1:**
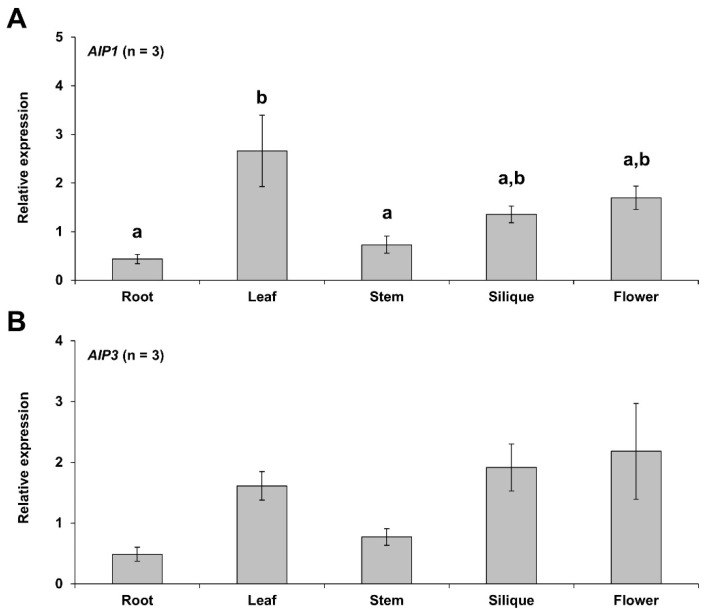
Expression patterns of *AIP1* and *AIP3* in various Arabidopsis tissues. RT-qPCR analysis of *AIP1* (**A**) and *AIP3* (**B**) relative transcript levels, performed using RNAs from various tissues. *ACT2* was used as an internal control. Error bars indicate standard errors; *n* = 30. Statistical significance was determined with one-way ANOVA followed by Tukey’s honestly significant difference (HSD) test; different letters denote significant differences (*p* ≤ 0.05).

*AIP1* and *AIP3* genes were expressed in all organs tested. *AIP1* showed a relatively higher expression level in leaves, while *AIP3* was highly expressed in flowers, although the expression patterns of these two genes were very similar (Figure 1A,B). Overall, *AIP1* and *AIP3* were more highly expressed in leaves, siliques, and flowers compared to other tissues, but *AIP3* did not show statistically significant differences in its expression levels across different organs.

### 2.2. Expression Patterns of AIP1 and AIP3 under Submergence and Hypoxic Environments

**Figure 2:**
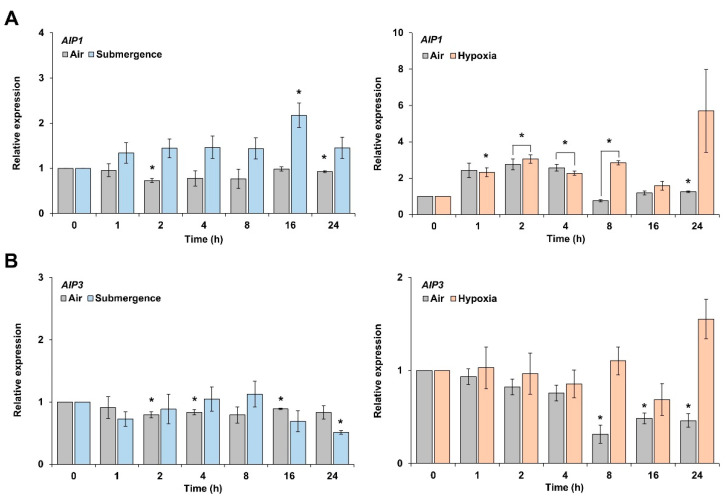
Expression patterns of *AIP1* and *AIP3* upon submergence or hypoxia. Seven-day-old seedlings were subjected to submergence or hypoxia in the dark. Control seedlings were maintained in normal air conditions in the dark and collected at the same time points. RT-qPCR analysis of *AIP1* (**A**) and *AIP3* (**B**) relative transcript levels from leaves. Error bars indicate standard errors. * *p* ≤ 0.05 vs. 0 h, as determined by Student’s *t*-test.

*AIP1* showed its strongest upregulation at 16 h after initiation of submergence, reaching levels ~2.5 times higher than air controls, before decreasing (Figure 2A).

Relative *AIP3* transcript levels rose steadily from 2 to 8 h after initiation of submergence before gradually decreasing at later time points (Figure 2B). Under a hypoxic environment, *AIP1* was expressed the highest at 24 h after the beginning of treatment, while *AIP3* was expressed the highest at 8 h, then dropped at 16 h, before rising again at 24 h (Figure 2A,B). Since *AIP3* did not show significant differences under submergence and hypoxia, we focused on *AIP1*.

### 2.3. Assessment of the Tolerance to Hypoxia of AIP1-Overexpressing Lines by Recovery-Survival Rates

**Figure 3:**
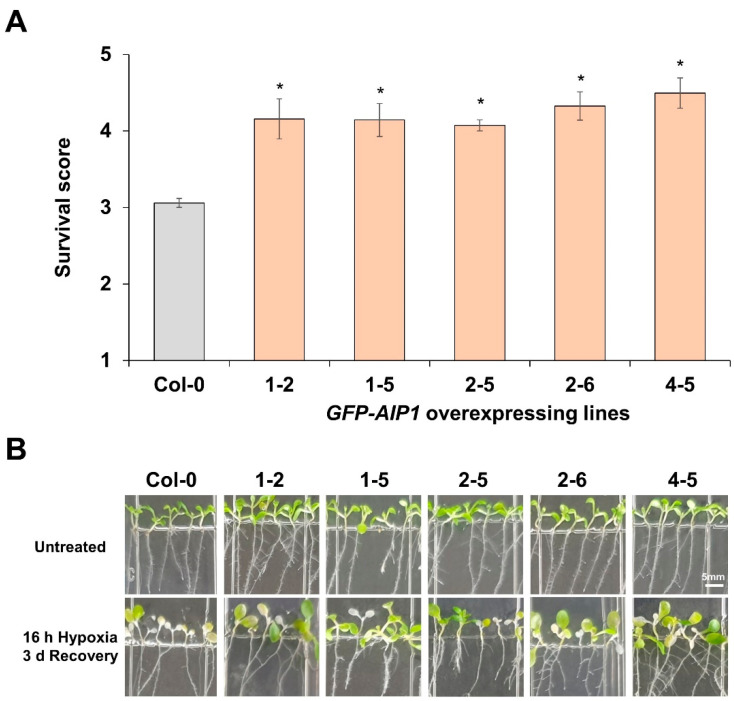
*GFP-AIP1* transgenic lines are more tolerant to hypoxic stress: (**A**) Survival scores of *GFP-AIP1*-overexpressing lines after a 16 h hypoxia treatment followed by a 3-day recovery period (Figure S2). Error bars indicate standard errors. * *p* ≤ 0.05 vs. Col-0, as determined by Student’s *t*-test. (**B**) Representative photographs of air- and hypoxia-treated (16 h) seedlings followed by a 3-day recovery period. Note the white seedlings for hypoxia-treated Col-0 plates.

To assess the contribution of *AIP1* to tolerance against low-oxygen environments, we generated transgenic Arabidopsis lines overexpressing *AIP1* (fused to *GFP*). To evaluate the tolerance of these transgenic plants to 16 h of hypoxic stress, we measured their survival rate following a 3-day recovery period. Five independent GFP-tagged transgenic lines exhibited higher survival rates than those of the wild-type non-transgenic control group (Figure 3). We selected line *GFP-AIP1-1-2* overexpressing *GFP-AIP1* for further characterization (Figure S1).

### 2.4. Assessment of the Tolerance to Hypoxia of an AIP1-Overexpressing Line by Electrolyte Leakage

**Figure 4:**
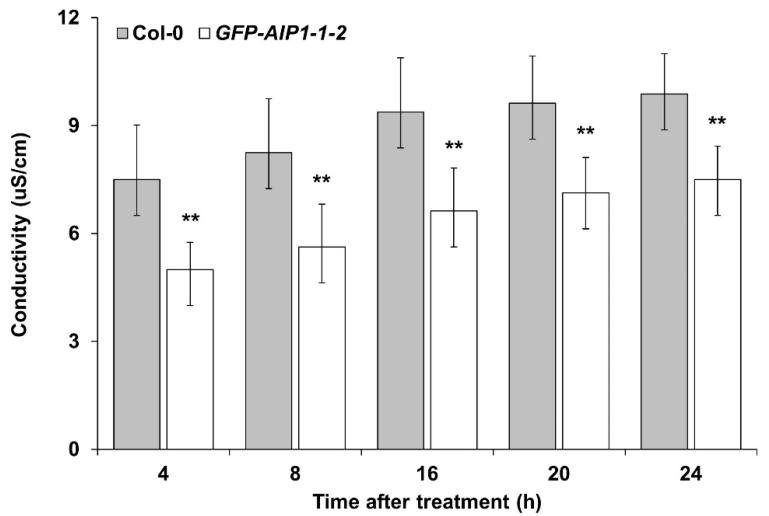
Electrolyte leakage from leaf disks between 4 and 24 h after hypoxic stress. Four-week-old plants were exposed to hypoxic conditions for 4 h. Electrolyte leakage of leaf disks was measured after 4–24 h of recovery under normoxia conditions. Error bars indicate standard error. ** *p* ≤ 0.01 vs. Col-0, as determined by Student’s *t*-test.

We next evaluated the electric conductivity of Col-0 and transgenic lines after hypoxic treatment. The electric conductivity of the *GFP-AIP1-1-2* line was significantly lower than that of the control Col-0 (Figure 4), in agreement with the higher survival score measured in the overexpression line (Figure 3).

### 2.5. Measurement of BCAA Contents in a GFP-AIP1-Overexpressing Line

**Figure 5:**
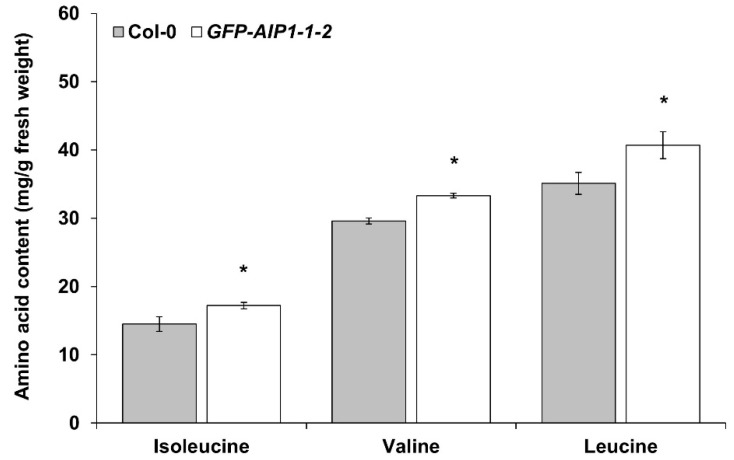
Measurements of BCAA contents in the *GFP-AIP1* overexpression line after hypoxic stress. Two-week-old plants were exposed to hypoxia for 4 h. The amounts of BCAAs were measured in leaves after hypoxic stress. Error bars indicate standard errors of two independent experiments, each with three technical replicates. * *p* ≤ 0.05 vs. Col-0, as determined by Student’s *t*-test.

Since AIP1 is a subunit of ALS, we determined the free amino acid contents with a focus on BCAAs, in Col-0 and the *GFP-AIP1-1-2* line after hypoxic treatment. BCAA contents (especially those of Val and Leu) were significantly (*p* ≤ 0.05) higher in the *GFP-AIP1-1-2* line relative to those in Col-0 (Figure 5).

## 3. Discussion

ALS is the enzyme catalyzing the first step in the biosynthesis of the BCAAs Val, Leu, and Ile [23,30]. As in bacteria and yeast (*Saccharomyces cerevisiae*), ALS in plants is composed of one catalytic and one regulatory subunit, forming a heterodimer whose activity is at least five times higher than homodimers of catalytic subunits [24,31]. A deeper understanding of the regulatory subunit is therefore very important to harness the catalytic power of ALS.

Roots and shoots may exhibit different tolerance mechanisms when faced with hypoxic stress [32]. In Arabidopsis, root and shoot responses to hypoxic stress are different [32,33]. In addition, several hypoxia-inducible genes, including a well-known hypoxic-response gene associated with fermentation, are abundantly expressed in roots [33,34]. For example, fermentation-related genes (*ALDEHYDE DEHYDROGENASE* [*ADH*], *PYRUVATE DECARBOXYLASE1* [*PDC1*], and *PDC2*) and several carbohydrate metabolism–related genes were more highly expressed in roots than in shoots after exposure to hypoxia for 1–24 h [10]. In the hypoxic state, Arabidopsis roots increase the expression of metabolism-related genes to overcome stress by creating a minimal level of ATP production [35]. However, *AIP1* and *AIP3* in this study were expressed to higher levels in the aboveground tissues and not in the roots. Considering that oxygen generated by active photosynthesis in leaves and stems mitigates the hypoxic state, we hypothesize that *AIP1* and *AIP3* are expressed mainly in oxygen-deficient tissues in leaves or stems, such as sieve tubes.

The inside of a sieve tube tissue is characterized by a relatively lower oxygen concentration than the surrounding tissues [36]. Measurements of the oxygen partial pressure using exudates in castor bean (*Ricinus communis*) revealed that that the oxygen concentration in the phloem was only 7% [36].

With a decrease in oxygen concentration, the content and current of sugar also decreased. There was a gradual increase in alanine, γ-aminobutyrate, methionine, and especially the BCAA Ile, as well as a gradual decrease in the carbon to nitrogen ratio, plus an increase in the ratio between succinate and malate in the phloem. These results suggest that metabolic functions and the functions of sieve tubes change adaptively with the oxygen concentration in the whole plant [36].

Flooding or submergence stress inevitably leads to hypoxic conditions by lowering intracellular oxygen partial pressure. *AIP1* expression increased after 16 h of submergence and after 24 h of hypoxia, and it was higher upon submergence or hypoxia stress compared to the control group (air treatment). However, *AIP3* displayed a lower expression level relative to the control group during the submergence treatment and followed a different pattern from that seen with *AIP1*. By contrast, *AIP3* exhibited a similar expression pattern as *AIP1* under hypoxic conditions, with a higher expression level after 24 h of hypoxia. *AIP1* and *AIP3* expression patterns were consistent under submergence and hypoxic conditions, prompting us to analyze the function of *AIP1*.

*AIP1*-overexpressing lines displayed better survival rates than the wild-type control during hypoxia-recovery tests (Figure 3), indicating that *AIP1* confers substantial resistance against hypoxic stress in Arabidopsis. We also confirmed the enhanced tolerance of *AIP1*-overexpressing lines to hypoxia by electrolyte leakage, as these transgenic lines showed less electrolyte leakage compared to the wild type (Figure 4). This improved tolerance may stem from higher BCAA contents, especially for Val and Leu (Figure 5).

BCAAs act as final receptor substitutes for electrons in fungi [29]. For instance, *Aspergillus nidulans* adapts to a hypoxic environment by employing a mechanism of NAD(P)H reoxidation to NAD(P)^+^ through the biosynthesis of BCAAs. NAD(P)^+^ can then be used for substrate-level phosphorylation to generate ATP. When applied to plants, this mechanism would produce NAD(P)^+^ as a by-product of BCAA biosynthesis, leading to a minimal pool of ATP that plants can utilize through substrate-level phosphorylation to survive. In this context, we conclude that activation of the ALS small subunit enhances the tolerance of Arabidopsis plants to hypoxia by increasing BCAA levels. Although it is inefficient compared to the production of ATP through respiration, plants can nevertheless access a supply of ATP to support some growth, even when in a hypoxic environment, ultimately contributing to plant tolerance to hypoxic stress. Further study on the precise roles of ALS small subunits in hypoxia tolerance will be required, as will studies on the undefined biological role of the ALS holoenzyme.

## 4. Materials and Methods

### 4.1. Plant Materials and Growth Conditions

Arabidopsis (*Arabidopsis thaliana*) accession Columbia (Col-0) was used as the wild type [37]. The seeds were stratified at 4 °C for 3 days in the dark after surface sterilization, planted in pots filled with sterilized Horticulture nursery Sunshine Mix No. 5 (Sun Gro Horticulture, Vancouver, BC, Canada), and cultivated in a culture room under a long-day photoperiod (16 h light/8 h dark) and 60% humidity at 24 °C [38].

### 4.2. RNA Extraction and cDNA Synthesis

Total RNA was extracted as described previously [39]. RNA was quantified on a Nanodrop DS-11 (DeNovix, Wilmington, NC, USA) and only high-quality RNA with a ratio between OD_260_/OD_280_ = 1.8–2.0 was used. The integrity and purity of the RNA were independently confirmed by electrophoresis on agarose gels. First-strand cDNAs were synthesized from 5 µg total RNA using a PrimeScript^TM^ 1st strand cDNA synthesis kit (TaKaRa, Kusatsu, Japan).

### 4.3. Tissue-Specific Expression of AIP1 and AIP3

Arabidopsis roots, stems, leaves, siliques, and flowers were collected for RNA extraction. Total RNAs were extracted from the roots and leaves of 7-day-old seedlings, 6-week-old stems, flowers from 8-week-old plants, and siliques from 9-week-old Arabidopsis plants [37].

RT-qPCR was conducted to check the organ-specific expression patterns of *AIP1* and *AIP3*. Primer Express v 3.0.1 (Applied Biosystems, Waltham, MA, USA) was used to design and build the primer pairs to amplify a PCR product between 100 and 150 bp. qPCR was performed with SYBR Premix Ex Taq^TM^ II(TaKaRa, Kusatsu, Japan) on a Thermal Cycler Dice Real Time instrument (TaKaRa, Kusatsu, Japan). The PCR conditions consisted of 40 consecutive cycles at 95 °C for 5 s and at 60 °C for 30 s.

### 4.4. Expression of AIP1 and AIP3 Genes upon Submergence and Hypoxia Conditions

For submergence treatment, Arabidopsis seedlings were first grown on plates with a half-strength Murashige and Skoog (MS) medium containing 0.5% (*w*/*v*) sucrose at 23 °C in a growth chamber for 1 week, before being moved to a glass box for submergence in the dark to minimize the production of oxygen from photosynthesis. After filling the glass box with distilled water to a height of 15 cm, the box was sealed and kept in the dark to maintain an oxygen-free submerged condition. At the same time, seedlings were incubated under normal air conditions without submergence as control. The seedlings were harvested for RNA extraction at seven time points (air: 0, 1, 2, 4, 8, 16, and 24 h; submergence: 1, 2, 4, 8, 16, and 24 h), as previously reported [38].

For each time point, the samples were divided into leaves and roots, and the leaves were used for RNA extraction. First-strand cDNAs were synthesized and subjected to qPCR as described above.

For hypoxic treatment, Arabidopsis were first grown on plates with half-strength MS medium containing 0.5% sucrose at 23 °C in a growth chamber for 1 week, before being transferred to a glass box for hypoxic treatment in the dark to minimize oxygen production from photosynthesis. The glass box was sealed and supplied continuously with 99.99% argon gas at a rate of 500 cubic centimeters (cc)/min to maintain hypoxic conditions. In parallel, seedlings were incubated under a normal air supply as a control. The seedlings were harvested for RNA extraction at seven time points (air: 0, 1, 2, 4, 8, 16, and 24 h; hypoxia: 1, 2, 4, 8, 16, and 24 h).

First-strand cDNAs were synthesized and subjected to qPCR as above.

### 4.5. Generation of AIP1-Overexpressing Arabidopsis Transformants

The cDNA of *AIP1* was cloned into an ENTRY vector (pENTR 2B) and then recombined with a DESTINATION vector (pK7WGF2) in-frame and downstream of the green fluorescent protein (*GFP*) coding sequence to generate a GFP fusion as previously reported [39]. The resulting constructs were introduced into Agrobacterium (*Agrobacterium tumefaciens*) strain GV3101 and transformed into Arabidopsis Col-0 plants by the floral dip method. Homozygous T_3_ lines were used for analyses after confirmation of constitutive overexpression of the genes.

### 4.6. Assessment of Hypoxia Tolerance of AIP1 Transformants by Measuring Recovery Rates

*AIP1* transgenic seeds were sown onto square plates containing a half-strength MS medium supplemented with 0.5% sucrose and then incubated for 1 week in a growth chamber at 23 °C. As a control, Col-0 seeds were sown next to transgenic seeds on the same plates. The plates were then transferred to a glass box and placed in the dark to minimize oxygen production from photosynthesis. Argon gas (99.99%) was continuously flowed into the sealed glass box at a rate of 500 cc/min to maintain a hypoxic state. After 16 h of hypoxic treatment, plates were transferred to a 23 °C growth chamber and allowed to recover in air at a normal oxygen concentration for 3 days. After 3 days of recovery, the degree of leaf damage of Col-0 seedlings and *AIP1* transformants was evaluated with the standard five-step scale [40]. Fifteen transgenic lines were subjected to the hypoxia-survival test, and five lines (1-2, 1-5, 2-5, 2-6, and 4-5) were selected for further analysis according to consistent survival rate.

### 4.7. Assessment of Tolerance to Hypoxia by AIP1 Transformants Based on Electrolyte Leakage Assay

Seeds from Col-0 and *AIP1* transformants were sown in pots filled with sterilized Horticulture nursery Sunshine Mix No. 5 (Sun Gro Horticulture) and cultivated for 4 weeks in a culture room under a long day photoperiod (16 h light/8 h dark) with 60% humidity at 23 °C. After exposure to hypoxia or air for 4 h, two leaf disks (6.5-mm diameter) were excised from each plant (one disk per leaf) using a cork borer. Immediately after excision, the leaf disks were floated onto 2 mL sterilized distilled water in a well of a 12-well plate. The plates were then re-exposed to air at a normal oxygen concentration for 4, 8, 16, 20, or 24 h. From each well, 100 µL exudate was collected and applied to an electrical conductivity meter (HORIBA, LAQUAtwin-EC-22, Kyoto, Japan) to measure conductivity [41].

### 4.8. Measurement of BCAA Contents in AIP1 Transformants

*AIP1* transgenic seeds were sown onto square plates containing a half-strength MS medium supplemented with 0.5% sucrose and incubated for 2 weeks in a growth chamber at 23 °C. As a control, Col-0 seeds were sown next to transgenic seeds on the same plates. The plates were then transferred to a glass box and placed in the dark to minimize oxygen production from photosynthesis. Argon gas (99.99%) was continuously flowed into the sealed glass box at a rate of 500 cc/min to maintain a hypoxic state. After 4 h of hypoxia or air treatment, the plant samples were divided into leaves and roots. Pulverized leaf tissues were hydrolyzed with 6 N HCL and then subsequently diluted with 0.02 N HCl. The Auto Amino Acid Analyzer (Hitachi, L-8900, Tokyo, Japan) was then used to quantify BCAA contents of the prepared samples.

## Data Availability

The data that support the findings of this study are available from the corresponding author upon reasonable request.

## Supplementary Materials

The following are available online at https://www.mdpi.com/article/10.3390/plants10112251/s1. Figure S1: Gene expression levels of *AIP1* in T_3_ plants determined by RT-PCR plants. *ACTIN2* was used as an internal control. Figure S2: Survival scores represent non-damaged, 25% damaged, half-damaged, 75% damaged, and dead (5, 4, 3, 2, and 1, respectively).
