# Supplementary figures and images for: AIP1, Encoding the Small Subunit of Acetolactate Synthase, Is Partially Responsible for Resistance to Hypoxic Stress in Arabidopsis thaliana
Source: Plants (Basel). 2021 Oct 22;10(11):2251. doi: 10.3390/plants10112251 (PMC8621687; doi:10.3390/plants10112251)

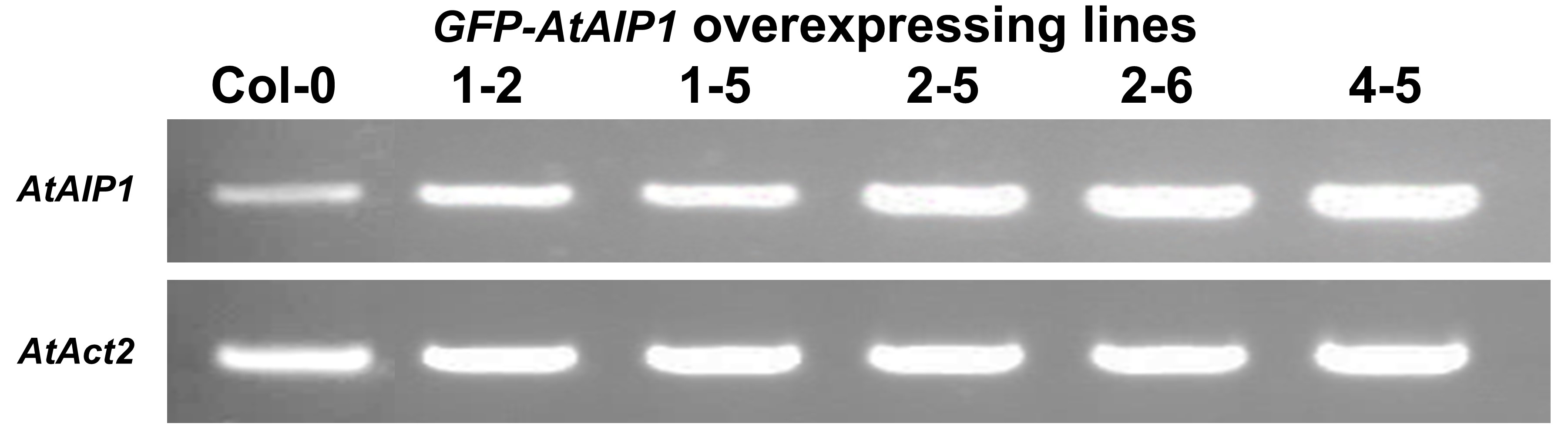

Supplement: Supplementary file 1 [file plants-10-02251-s001.zip › Figure S1.jpg]

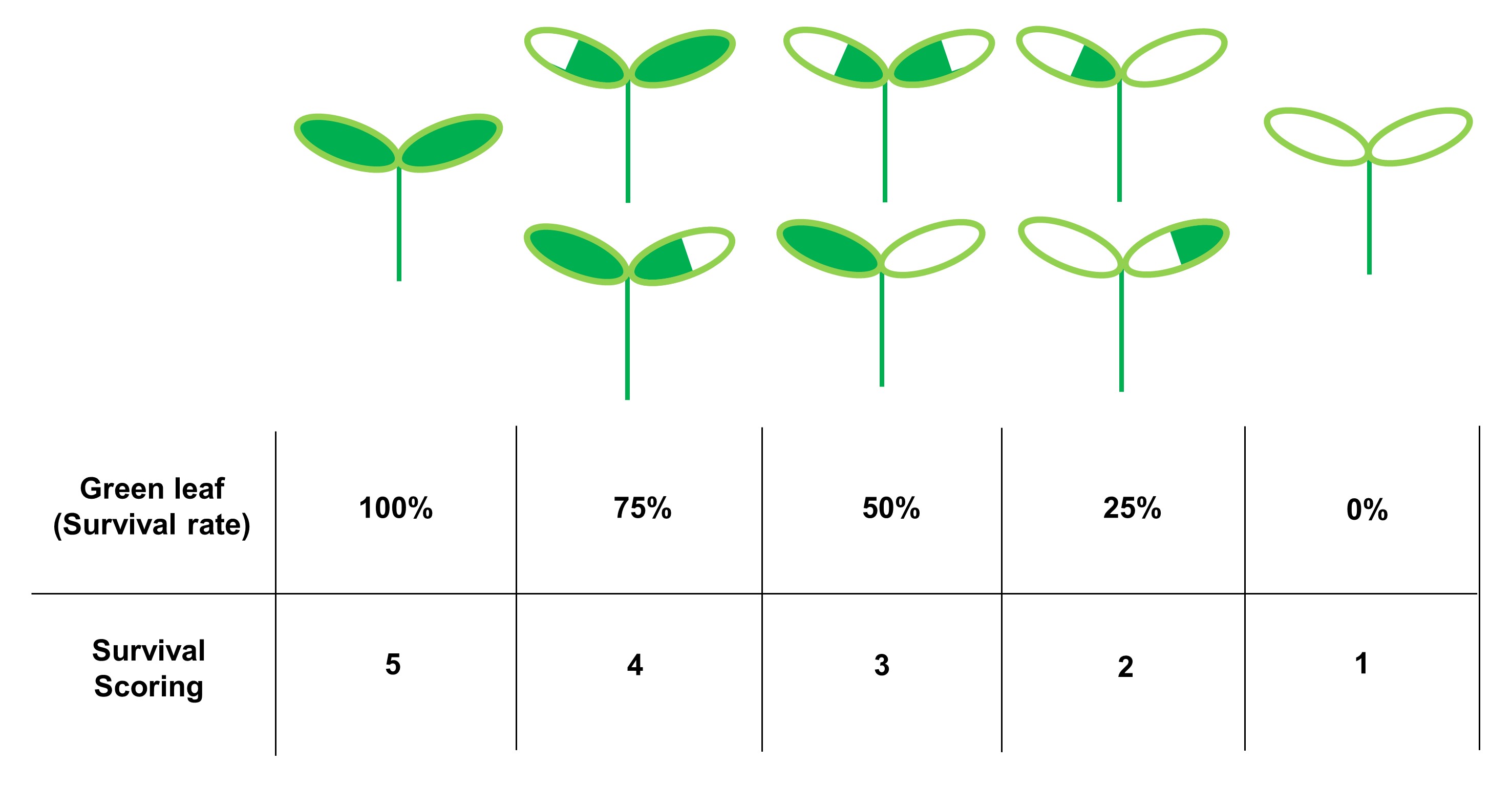

Supplement: Supplementary file 1 [file plants-10-02251-s001.zip › Figure S2.jpg]
